# A patients’ view of OA: the Global Osteoarthritis Patient Perception Survey (GOAPPS), a pilot study

**DOI:** 10.1186/s12891-020-03741-0

**Published:** 2020-11-07

**Authors:** Marianna Vitaloni, Angie Botto-van Bemden, Rosa Sciortino, Xavier Carné, Maritza Quintero, Pedro Santos-Moreno, Rolando Espinosa, Oscar Rillo, Jordi Monfort, Francisco de Abajo, Elizabeth Oswald, Marco Matucci, Patrick du Souich, Ingrid Möller, Montserrat Romera Baures, Arlene Vinci, Deborah Scotton, Marco Bibas, Guy Eakin, Josep Verges

**Affiliations:** 1 Osteoarthritis Foundation International (OAFI), Barcelona, Spain; 2 Arthritis Foundation, Atlanta, USA; 3 De los Andes University, Merida, Venezuela; 4 Hospital Clinic, Barcelona, Spain; 5 Biomab - Centro de Arthritis Reumatoide, Bogota, Colombia; 6 Instituto de Medicina Nacional de Rehabilitación, Ciudad de Mexico, Mexico; 7 Hospital. I. Pirovano, Buenos Aires, Argentina; 8 Hospital del Mar, Barcelona, Spain; 9 University of Alcalá (IRYCIS), University Hospital Principe de Asturias, Madrid, Spain; 10 University of Florence, Florence, Italy; 11 University of Montreal, Montreal, Québec Canada; 12 Institut Poal, University of Barcelona, Barcelona, Spain; 13 Hospital de Bellvitge, Hospitalet de Llobregat, Spain

**Keywords:** Quality of life, Patient perception, Osteoarthritis, Global survey, Patient organizations

## Abstract

**Background:**

Globally, osteoarthritis (OA) is the third condition associated with disability. There is still poor treatment in OA but science holds the key to finding better treatments and a cure. It is essential to learn what’s important to patients from them to implement the most effective OA management. The OA Patients Task Force, conducted the Global OA Patient Perception Survey (GOAPPS)-the first global survey made by patients to analize the quality of life (QoL) & patient perceptions of care. The goal was to collect data on OA patients’ perception of OA to understand patients’ needs and expectations to improve OA management.

**Methods:**

Observational, cross-sectional study by online survey data collection from six countries, translated into three languages. The questionnaire was comprised of 3 sections: patient demographics and clinical symptomology characteristics; relationship with physicians: perception of attention, treatment, and information provided; and OA impact on daily activity and QoL. The results of the survey were evaluated using the Limited Data Set. The survey results were analyzed using descriptive statistics to characterize the patients’ answers. Additionally, Cronbach’s alpha was calculated to determine internal consistency validity.

**Results:**

A total of 1512 surveys were completed in 6 countries. 84.2% of respondents reported pain/tenderness and 91.1% experienced limitations to physical activities. 42.3% of patients were not satisfied with their current OA treatment. 86% had comorbidities, especially hypertension, and obesity. 51.3 and 78% would like access to additional drug or additional non-drug/non-surgical treatments respectively. 48.2% of patients perceived their QoL to be affected by OA. The Cronbach’s alpha was 0.61.

**Conclusions:**

OA has a significant impact on patients’ daily activities and their desire to play an active role in managing this disease. Patients are seeking additional treatments, especially no pharmacological/no surgical treatments stressing the need for investing in clinical research, implementing OA preventive measures, and managing interventions to improve the healthcare value chain in OA.

**Supplementary Information:**

**Supplementary information** accompanies this paper at 10.1186/s12891-020-03741-0.

## Background

Globally, osteoarthritis (OA) is the most common musculoskeletal disorder and it is associated with pain, disability, and quality-adjusted life-year losses [1–4]. From 1990 to 2013, the trend of Years Lived with Disabilities (YLD) in OA increased 75%, being OA the third most rapidly rising condition after diabetes and dementia [1]. Worldwide, around 300 million people suffer from OA. Of these, more than 40 million adults live in Europe, and over 30 million in the United States of America (USA). In Latin America, the numbers of adults with OA are only partially available [1, 5, 6]. Adults afflicted with OA display different degrees of disability, ranging from mild and intermittent pain, with minimal difficulty in performing daily activities, to chronic pain with progressive structural damage and loss of function. Greater disability is often associated with a decrease in mental health and an increase in mortality when a person can no longer walk or live independently [1].

Currently, there is no cure for OA. However, there is increasing interest in developing OA interventions that improve physical health and quality of life (QoL) while reducing opioid or NSAID abuse and associated comorbidities [7–13]. To accomplish this, a consensus about the outcomes that are relevant to OA patients, comorbid populations, and other key stakeholders in OA requires listening to patients’ opinions and experiences. Learning what is important to patients from the patients themselves is essential to create a core outcome set to bridge the gap between the patients’ needs and the current OA management paradigms.

Integrated people-centered health services, as defined by the World Health Organization, implies putting the comprehensive needs of people and communities, not only the conditions as such, at the center of health systems, and empowering people to have a more active role in their health [14]. To support OA patients, the Osteoarthritis Foundation International (OAFI) (Barcelona, Spain) and the Arthritis Foundation (Atlanta, USA) with participation from important organizations fighting against rheumatic disease worldwide such as the Panamerica League against Rheumatism (PANLAR) created the OA Patients Task Force. This task force is a global alliance working in the fight against OA and representing about 150 million patients.

In 2018, the OA Patients Task Force developed the Global OA Patient Perception Survey (GOAPPS). This was the first international survey developed by patients' organizations to investigate OA patients’ perceptions of QoL across many languages and nations/cultures using the same questionnaire. This was a pilot study aims to collect information on OA patients’ perceptions regarding the impact of the disease in their lives to help all stakeholders involved in OA healthcare and management to better understand patients’ perceptions, to address patient needs more effectively in terms of prevention, research, and management [15].

## Methods

This observational, cross-sectional study collected data through an online survey in six countries where member organizations of the OA Patients Task Force were based: Colombia, Spain, Italy, Mexico, the USA, and Venezuela. It was a hypothesis generating research aim to collect a set of data to decipher the relationship and patterns existing between different aspects of patient’s perceptions of QoL.

Patients were required to meet the following inclusion criteria to be eligible for this study: (i) being residents of one of the participant countries, (ii) being older than 18 years, and (iii) reporting to have been diagnosed with OA by their physician.

### Questionnaire development

The questionnaire was developed by researchers collaborating with the OA Patients Task Force and included expert patients, rheumatologists, clinical pharmacologists, epidemiologists and, patient advocates.

The first version of the questionnaire was written in English, then was translated into Spanish and Italian using a specific protocol of forward translation, back translation, and resolution. The translation process was supervised by the Survey Coordinator designated in each region.

During forward translation, a health professional familiar with relevant terminology, fluent in English but whose mother tongue was the primary language of the target culture, translated the survey from English to the local language. The focus was on conceptual rather than literal translation and the use of natural and acceptable language for the broadest audience. During back translation, a different translator who was a native English speaker translated the survey from the local language to English.

The forward translator, back translator, and Survey Coordinator met to resolve any concerns or discrepancies in the forward translated language. The Survey Coordinator sent the translation documentation to international Project Coordinators to ensure coordination of survey language across participating countries.

The final questionnaire was divided into 3 sections with an acknowledgment statement that read: “By entering the survey, I indicate that I have read the information provided and agree to participate.” The first section of the survey included patient demographics and clinical characteristics questions. The second section of the survey focused on patient relationships with physicians and explored the personal perception of attention, treatment, and information received. In the last section of the survey, patients were asked to evaluate their QoL. Finally, two questions were added to explore the patients’ interest in the survey results and their willingness to participate in a future survey on OA.

The OAFI Patients Committee, composed of 15 volunteers OA patients, evaluated the ethical aspects of the final version of the questionnaire. The members of the OAFI Patients Committee approved the questionnaire compliant with patient use.

Cronbach’s alpha was used to measure the reliability of the global questionnaire. The Cronbach’s alpha was 0.61. Cronbach’s alpha value of 0.60 is accepted by the scientific community in the case of exploratory research [16]. Newly developed instruments can be accepted with an alpha value of 0.60 [17].

### Survey execution

The engagement with patients occurred in two stages: 1) a pilot test and 2) the online administration.

During the pilot test, the local Survey Coordinators administrate the questionnaire to 10 volunteer patients who met the inclusion criteria to evaluate the questionnaire burden, acceptability, and comprehensibility. As a result, some questions were modified to avoid unfamiliar terms or removed to shorten or avoid repetition (Additional file 1).

The final survey was administered by the Arthritis Foundation using the online secured Qualtrics platform from June to November 2018, a survey software allowing to conduce personalized surveys. The local Survey Coordinator administrated the survey in each country through collaboration with local organizations that promoted access to the survey webpage using social media, brochures, or other promotional materials.

The results of the survey were evaluated using the Limited Data Set. The survey results were analyzed using descriptive statistics to characterize the patients’ answers using the IBM SPSS® software.

This survey was considered a pilot study.

## Results

### Demographic and clinical data

A total of 1683 patients entered the online survey. One thousand five hundred twelve entries were considered correctly completed. The final sample includes patients from the USA, Spain, Colombia, Venezuela, Mexico, Italy. The demographic data of patients surveyed are shown in Table 1.

**Table 1:** Demographic data from completed surveys

	%	No
Questionnaires completed		1512
Sex		
Male	14.5%	219
Female	85.5%	1293
Total	100%	1512
Age		
18–39	1.7%	25
40–59	26.3%	397
60–79	65.5%	991
> 80	6.5%	98
N/A	0.1%	2
Total	100%	1512
Primary country of origin		
USA	82,2%	1243
Venezuela	11.4%	172
Spain	4.0%	60
Colombia	1.9%	29
Mexico	0.4%	6
Italy	0.1%	2
TOTAL	100%	1512

**Fig. 1:**
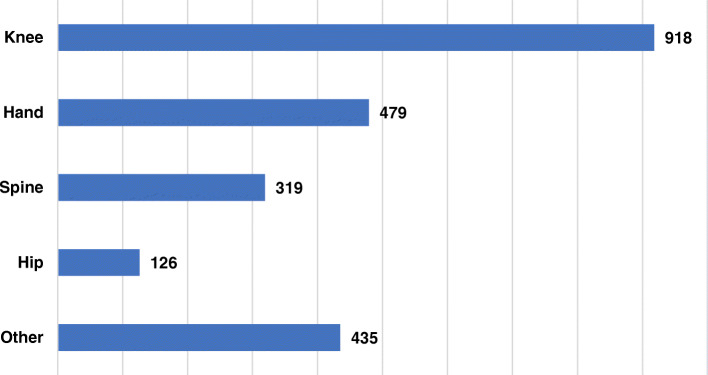
Joints with OA as reported by patients. Patients could select all the options that apply. Each column represents the localization(s) of OA as diagnosed by their medical doctor. Number of patients answering this question = 1512. Number of answers = 2277

The survey results indicated the majority of patients were diagnosed with knee OA (60.7%; 918 patients out of 1512). This was followed by hand OA (31.7%; 479 patients out of 1512), spine OA (21.1%; 319 patients out of 1512), and hip OA (8.3%; 126 patients out of 1512) (Fig. 1; Additional file 2). Also, 435 (28.8%) of patients reported having OA in other joints, mainly feet, shoulder, and ankle (Additional file 3). The survey did not limit the number of OA diagnoses so that patients could select all the OA locations that applied to their case.

**Fig. 2:**
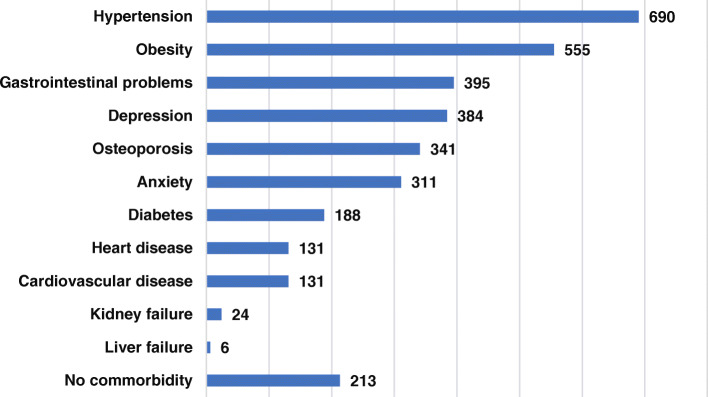
Patients’ comorbidities as diagnosed by a medical doctor. Patients could select all the options that apply. Number of patients answering this question = 1512. Number of answers = 3369

Regarding comorbidities, 86% of respondents reported being diagnosed with one or more comorbidities: 27.3% reported having one comorbidity, while 24.6% and 16.5% reported having two and three comorbidities respectively. Arterial hypertension (45.6%) and obesity (36.7%) were the most commons comorbidities reported. Gastrointestinal problems (26.1%) were the third most reported comorbidity. Mental health problems, such as depression and anxiety, had a high prevalence, as reported by 25.6 and 20.6% of respondents, respectively. Osteoporosis was associated with OA in 22.6% of patients. Furthermore, 12.4% of OA patients suffered from diabetes. (Fig. 2; Additional file 2).

**Fig. 3:**
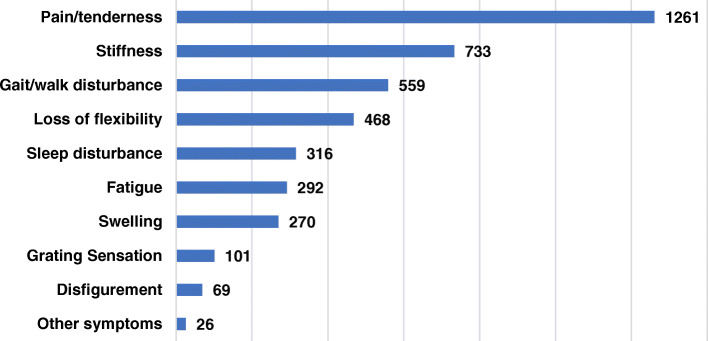
Clinical symptoms, which have the most significant impact on patients’ daily life as reported by patients. Patients can select up to three options. Number of patients answering this question = 1498. Number of answers = 4095

Regarding OA symptoms, patients could select up to three options. The combination of pain/tenderness, stiffness, and gait/walk disturbance was the commonest symptom reported that most significantly impacted their daily life. 84.2% of patients declared suffering for pain/tenderness, 48.9% for stiffness, and 37.7% of gait/walk disturbance. Also, loss of flexibility, sleep disturbance, fatigue, and swelling were reported as high prevalence symptoms in OA patients (Fig. 3; Additional file 2).

### Patient-reported perceptions of patient-physician relationships

When patients were asked about their relationships with their doctors, about 7 in 10 patients (70.8%) agreed or strongly agreed with the statement that their doctors “understand” them when they describe their OA symptoms (versus 13% who disagreed or strongly disagree) and almost 6 in 10 patients (58.3%) agreed or strongly agreed their doctor adequately explained their OA diagnosis and what it represented to them (versus 22.1% disagreed or strongly disagreed). While a little more than half of the patients (53.3%) stated that they understood their OA treatment options and the associated risks, almost one-quarter of patients (24.6%) stated they did not understand their treatment options and associated risks. Conversely, more than 4 in 10 patients (42.3%) were not satisfied with their current OA treatment, while only about one-quarter of patients (26.8%) stated they were satisfied with their current OA treatment (Additional file 4).

Regarding the possibility of access to additional treatment, the highest proportion of patients (78%) stated they would like to have access to additional non-drug/non-surgical treatments for their OA. This was followed by a little over half of the patients (51.3%) who stated they would like to have access to additional drug treatment options for their OA and more than one-third of patients (35%) who would like access to additional surgical treatment options. About 1 in 5 patients (18.9%) were not interested in additional drug treatment options and more than 1 in 3 patients (36%) were not interested in additional surgical treatment options.

### Patients’ perception of QoL

**Fig. 4:**
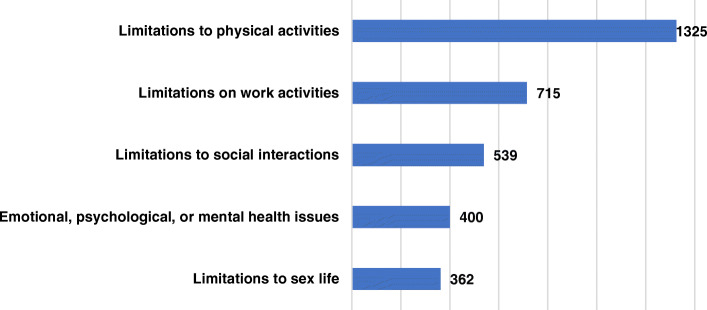
Limitations or issues experienced by patients due to OA. Number of patients answering this question = 1455. Number of answers = 3341

Regarding the limitations experienced due to OA, 91.1%% of the patients reported limitations in their everyday life physical activities, followed by 49.1% of patients reporting limitations to their work activities, and 37% in social interactions. OA has emotional, psychological, or mental health consequences in almost one-third of respondents and limited their sex life in 24.9% of patients. (Fig. 4; Additional files 2 and 5).

About half of the patients (51.7%) reported being satisfied with their QoL while the other half were either not satisfied or not sure about their satisfaction with their QoL (48.2%). When asked how their QoL would be if their OA signs and symptoms were eliminated, 94.2% of respondents said that their QoL would be good or very good.

Finally, 74% of patients said that they would like to be informed about the results of this study and 79% would like to participate in future research on OA.

## Discussion

OA is one of the most prevalent diseases affecting people globally and is a leading cause of pain and disability among adults [18, 19]. Despite being such a widespread and severe disease, it usually associates with the false belief that it is a normal part of aging and that patients have to accept and live with it. This false myth, for example, makes OA patients least likely to receive exercise therapy and weight loss advice as recommended by international clinical guidelines as a first-line treatment for OA [20].

Empowering patients to have a more active role in their health and engaging them in research are key aspects to ensure the effective implementation of interventions aimed at improving QoL of patients and, consequentially, the current OA management strategies. Patients should be considered as another stakeholder within the health system and patients’ organizations should be considered as another healthcare agent, present in real decision making, to make patient voice be heard. It is necessary to know which outcomes are relevant for patients to ensure their participation and satisfaction.

The OA Patients Task Force, an international alliance of patients’ organizations, has been working since 2016 on independent projects to make OA more visible and to empower patients. The GOAPPS survey was a pilot study designed to collect and analyze data on OA patients’ perceptions regarding their health conditions, OA care, and to explore the impact of OA on daily functioning and QoL. The results aimed at providing a portrait of patients’ perceptions of OA to provide a baseline to take better quality performance in the future. As a pilot study, it aimed to capture the necessary information to carry out a large-scale survey able to compare cross-cultural and cross-national data. Also, it was a proof of concept study designed to prove the strength and capacity of patients and their organizations to the scientific community.

The results of this pilot study confirmed that OA can be considered a gendered disease being more prevalent among women [21–24]. Although, the fact that the majority of respondents were women can also be interpreted as women being more active and willing to participate in these types of surveys. The prevalence of OA increases with age in line with our results showing that the age range with the highest prevalence of OA was 65 to 74 years. However, almost a third of participants were 40 to 59 years indicating that OA also affects younger groups of people like pre-menopausal women, athletes, or injured people [25]. Finally, the majority of the respondents were from the USA. This could be a consequence of the different computer literacy existing among the different countries that participated in the survey. The data could also reflect the importance and strength that patient organizations have in each country which can associate with the structure of national health systems.

The survey results revealed that OA patients are affected by multiple comorbidities, especially arterial hypertension and obesity. Notably, half of this population suffers from depression and/or anxiety. The presence of comorbidities increases the frequency of physical disability in patients with OA, and the impact on the QoL of patients of the combination is greater than that expected for OA alone or each isolated disease [18]. Comorbidities have been reported to be more frequent in patients with OA compared with other diseases and to lead to greater deterioration of physical functions and QoL. OA increases cardiovascular mortality [26], worsens the prognosis of arthroplasties, and reduces the range of possibilities of pharmacological treatment due to the incompatibility of prescribed drugs to alleviate joint pain and treat comorbidities [4]. Finally, our results revealed a high prevalence of gastrointestinal problems which may be a consequence of the high use of NSAIDs, the oral OA treatment of choice in the majority of the cases that are related to gastrointestinal adverse effects [27].

More than three-quarters of these patients (78%), who are probably already polymedicated, ask for access to additional non-drug/non-surgical treatments for their OA. Non-pharmacological therapies such as physical activity or nutritional programs have been recommended in clinical guidelines and reported to have a positive effect on the health status and QoL of OA patients [7, 28]. It has been shown that exercise therapy may postpone total joint replacement [29]. Physical and occupational therapy-related interventions have proven to reduce pain in patients with hand OA [30, 31]. Additionally, self-management programs have shown to improve mental health and social connectedness, thereby improving many aspects of OA patients’ lives [32–34].

The high percentage of respondents asking for access to these interventions is striking considering the existence of such extended literature describing effective interventions for OA management. This may highlight an existing gap between the theory on how to improve OA management and the reality patients have to face when living with their disease. Health systems should invest more in implementing health promotion and intervention programs in OA while partnering with patients’ organizations. Similarly, educational programs should be promoted, both for health professionals and patients. Only 58.3% of respondents said their doctors explained adequately their OA diagnosis and only 53.3% understood their OA treatment options. Educational interventions are extremely important tools able to improve patients’ ability to self-manage their chronic diseases hence improving their QoL [35, 36].

Almost all patients reported limitations in physical and work activities, as well as enormous limitations in their personal life associated with severe symptomatology (i.e., pain, stiffness, loss of flexibility among others). Pain and other OA-related symptoms can be reduced by rehabilitation programs focused on alleviating pain and maintaining or improving physical and psychological function. Rehabilitation is widely recommended as first-line treatment for OA in evidence-based clinical guidelines [32, 37–39], as they are safer and, in many cases, more effective at reducing pain than the best established pharmacological interventions. Also, regular exercise is considered to be a core treatment for OA and it is universally recommended amongst treatment guidelines for all individuals with OA [40, 41].

When only 26.8% of respondents report being satisfied with their current treatment plan, it is not surprising that more than half of the respondents would like to have access to additional drug treatments, a request which underlines the urgent need for new medications for OA. Currently, there is no cure for OA and pharmacological treatments can help to relieve symptoms or delay the progression of the disease. However, many of these drugs cannot be used for a long time due to their adverse effects and incompatibility with medications used for OA- associated comorbidities [26, 42–45]. Furthermore, it has been previously reported that OA patients are concerned about possible side effects of medication [46]. This evidence, in association with our results, highlights the need for investment in research for new and more active OA pharmacological treatments.

In this study, only 51.7% of patients reported having a good QoL; whereas 48.2% were either not satisfied with their QoL or not sure about their satisfaction with their QoL. It has to be noted that elderly people can have, in general, lower expectations in terms of QoL than young patients [47]. The age range of our study population was older and this may have been reflected in this survey’s QoL responses. Additionally, the false conception that OA is a natural age-related condition may lower the expectations of QoL of OA patients while highlighting the need for patient education programs which may have an impact on OA patients’ perception. When patients were asked how they would evaluate their QoL if OA was eliminated, almost 95% of respondents said they would be more satisfied. This data demonstrates the impact of OA on the QoL of people affected by this disease and the urgent need for improvement in OA management strategies.

### Limitations of the study

The results of this pilot study present limitations which must be acknowledged. The data used in the analyses were based on patient self-reports, without clinical verification of an OA diagnosis, and thus are subject to the biases that are inherent to this type of data survey. Also, online questionnaires can be associated with gender and age-related biases, as women may be more prompt in using this kind of technology and elderly people may find it difficult to respond to them.. There is a huge difference in the number of surveys collected in each of the participant countries that could reflect cultural perceptions of this type of survey and/or technology. Caution should be taken when interpreting these findings, as there is a clear predominance of surveys answered by patients from the USA.

## Conclusion

The results of this pilot study represent an important stepping stone to gain insight into the needs and priorities of OA patients to shape the health care process on the patient’s view. The results highlight the severe impact of OA on patients’ life due to associated limitations, symptoms, and comorbidities. Importantly, the vast majority of patients expressed an interest in gaining access to non-drug/non-surgical treatment. Health promotion and self-management strategies addressing unhealthy weight and low levels of physical activity may improve the health conditions of patients as well as educational programs incorporating the patient expert role. Also, patients are requesting more pharmacological treatments as an alternative to the symptomatological drugs currently available on the market which can only alleviate OA symptoms and may be associated with unwanted adverse effects. This portrait of patients’ perceptions of OA can provide a baseline to evaluate better quality performance in the future. Starting with these results and considering the study limitations, the OA Patients Task Force aims to develop a new international survey that will enable them to reach a larger population and collect more data on the patients’ population characteristics (i.e. socioeconomic), perception and patient needs which can allow to the performance of population distribution analyses and the ability to understand the specific needs and characteristics of different demographic and social groups. Depth analysis of the reasons behind the patients’ needs may contribute to the design of personalized strategies for OA management and treatment.

## Supplementary Information

**Additional file 1.** Global Osteoarthritis Patient Perception Survey (GOAPPS) questionnaire. English version.

**Additional file 2.** Percentages as calculated on the total number of answers and on the total number of respondents.

**Additional file 3.** Figure showing the joints reported by participants in the category “other”. Patients could report OA in joints other than the options offered as an answer (knee, hip, spine, hand). Each column represents the localization(s) of OA as diagnosed by their medical doctor.

**Additional file 4.** Table showing participants’ responses to questions related to the causes of OA and their relationship with their doctor.

**Additional file 5.** Table reporting the limitations experienced due by OA as reported.

## Data Availability

The datasets used and/or analysed during the current study are available from the corresponding author on reasonable request.

## Abbreviations

GOAPPS1: Global OA Patient Perception Survey
NSAIDs: NonSteroidal Anti-Inflammatory Drugs
OA: Osteoarthritis
OAFI: Osteoarthritis Foundation International
PANLAR: Panamerica League against Rheumatism
QoL: Quality of Life
USA: United States of America
YLD: Years Lived with Disabilities
