# Supplementary material for: A patients’ view of OA: the Global Osteoarthritis Patient Perception Survey (GOAPPS), a pilot study
Source: BMC Musculoskelet Disord. 2020 Nov 7;21:727. doi: 10.1186/s12891-020-03741-0 (PMC7648975; doi:10.1186/s12891-020-03741-0)
Supplement: Supplementary file 1 — Additional file 1. Global Osteoarthritis Patient Perception Survey (GOAPPS) questionnaire. English version. [file 12891_2020_3741_MOESM1_ESM.docx]

**Additional file 1.**

Global Osteoarthritis Patient Perception Survey (GOAPPS) questionnaire

English version

*Please select your country of primary residence from the menu below:*

Italy

Spain

United States

Other, please specify:

Are you 18 or older?

Yes

No

What year were you born?

1. Have you ever been diagnosed with osteoarthritis? In other words, has a medical doctor examined you and, based on the exam results, told you that you have osteoarthritis?

Select one:

Yes

No

1. In which joint(s) do you have osteoarthritis? Mark all that apply:

Knee

Hip

Spine

Hand

Other (please specify the location)

1. Have you been diagnosed with any of the following conditions? That is, has a medical doctor ever told you that you had one or more of the conditions listed below? Mark all that apply:

Obesity

Diabetes

Heart disease/Cardiovascular disease

Hypertension

Osteoporosis

Depression

Anxiety

Kidney failure

Liver failure

Gastrointestinal problems

1. What is your biological sex? Select one:

Male

Female

1. Of all the clinical symptoms you have experienced because of osteoarthritis, which do you consider to have the most significant impact on your daily life? Check up to three:

Pain / tenderness

Swelling

Stiffness

Grating sensation

Loss of flexibility

Gait/walk disturbance

Sleep disturbance

Fatigue

Disfigurement

Other symptoms not mentioned

1. Which of the following limitations or issues have you experienced due to osteoarthritis? Select all that apply:

Limitations to physical activities

Limitations to social interactions

Limitations to work activities

Limitations to sex life

Emotional, psychological, or mental health issues

*In this section of the survey, you will read a series of statements. For each statement, indicate your level of agreement.*

1. I understand the common causes of osteoarthritis. Select one:

Strongly disagree

Disagree

Neither agree nor disagree

Agree

Strongly agree

1. My doctor understands me when I describe the symptoms I experience due to osteoarthritis. Select one:

Strongly disagree

Disagree

Neither agree nor disagree

Agree

Strongly agree

1. My doctor adequately explained my osteoarthritis diagnosis to me. Select one:

Strongly disagree

Disagree

Neither agree nor disagree

Agree

Strongly agree

1. I understand my osteoarthritis treatment options and the associated risks of each option. Select one:

Strongly disagree

Disagree

Neither agree nor disagree

Agree

Strongly agree

1. I am satisfied with my current osteoarthritis treatment plan. Select one:

Strongly disagree

Disagree

Neither agree nor disagree

Agree

Strongly agree

1. I would like access to additional drug treatment options for my osteoarthritis (for example: glucosamine, chondroitin, NSAIDs, analgesics such as ibuprofen or acetaminophen, hyaluronic acid, corticosteroids)

Strongly disagree

Disagree

Neither agree nor disagree

Agree

Strongly agree

1. I would like access to additional surgical treatment options for my osteoarthritis (for example: total knee replacement, arthroscopy). Select one:

Strongly disagree

Disagree

Neither agree nor disagree

Agree

Strongly agree

1. I would like access to additional non-drug /non-surgical treatment options for my osteoarthritis (for example: weight management, injury prevention, exercise programs). Select one:

Strongly disagree

Disagree

Neither agree nor disagree

Agree

Strongly agree

*In this section of the survey, please read each question, assess your feelings, and select the option that gives the best answer for you for each question.*

1. How would you rate your quality of life?

Very poor

Poor

Neither poor nor good

Good

Very good

1. If your osteoarthritis were eliminated, how would you rate your quality of life? Select one:

Very poor

Poor

Neither poor nor good

Good

Very good

1. I am interested in receiving cumulative survey results

Yes

No

1. I am interested in participating in future surveys

Yes

No
