# Supplementary material for: A patients’ view of OA: the Global Osteoarthritis Patient Perception Survey (GOAPPS), a pilot study
Source: BMC Musculoskelet Disord. 2020 Nov 7;21:727. doi: 10.1186/s12891-020-03741-0 (PMC7648975; doi:10.1186/s12891-020-03741-0)
Supplement: Supplementary file 2 — Additional file 2. Percentages as calculated on the total number of answers and on the total number of respondents. [file 12891_2020_3741_MOESM2_ESM.docx]

**Additional file 2**

Percentages as calculated on the total number of answers and on the total number of respondents.

1. Joints with OA as reported by patients

| **Joint** | **N** | **Percentage (n=2277)*** | **Percentage of cases (n=1512)**** |
| --- | --- | --- | --- |
| *Knee* | 918 | 40,3% | 60,7% |
| *Hand* | 479 | 21,0% | 31,7% |
| *Spine* | 319 | 14,0% | 21,1% |
| *Hip* | 126 | 5,5% | 8,3% |
| *Others* | 491 | 19,1% | 28,8% |
| *TOTAL* | 2277 | 100% | 150,6% |

*Total answers

**Number of respondents

2. Patients' comorbidities as diagnosed by a medical doctor.

| **Comorbidity** | **N°** | **Percentage**  **(n=3369)*** | **Percentage of cases (n=1512)**** |
| --- | --- | --- | --- |
| *Hypertension* | 690 | 20,5% | 45,6% |
| *Obesity* | 555 | 16,5% | 36,7% |
| *Gastrointestinal problems* | 395 | 11,7% | 26,1% |
| *Depression* | 384 | 11,4% | 25,4% |
| *Osteoporosis* | 341 | 10,1% | 22,6% |
| *Anxiety* | 311 | 9,2% | 20,6% |
| *Diabetes* | 188 | 5,6% | 12,4% |
| *Heart disease* | 131 | 3,9% | 8,7% |
| *Cardiovascular disease* | 131 | 3,9% | 8,7% |
| *Kidney failure* | 24 | 0,7% | 1,6% |
| *Liver failure* | 6 | 0,2% | 0,4% |
| *No commorbidity* | 213 | 6,3% | 14,1% |
| *TOTAL* | 3369 | 100,0% | 222,8% |

*Total answers

**Number of respondents

3. Clinical symptoms, which have the most significant impact on patients’ daily life as reported by patients

| **Symptom** | **N°** | **Percentage (n=4095)*** | **Percentage of cases (n=1498)**** | |
| --- | --- | --- | --- | --- |
| *Pain/tenderness* | 1261 | 30,8% | | 84,2% |
| *Stiffness* | 733 | 17,9% | | 48,9% |
| *Gait/walk disturbance* | 559 | 13,7% | | 37,3% |
| *Loss of flexibility* | 468 | 11,4% | | 31,2% |
| *Sleep disturbance* | 316 | 7,7% | | 21,1% |
| *Fatigue* | 292 | 7,1% | | 19,5% |
| *Swelling* | 270 | 6,6% | | 18,0% |
| *Grating Sensation* | 101 | 2,5% | | 6,7% |
| *Disfigurement* | 69 | 1,7% | | 4,6% |
| *Other symptoms* | 26 | 0,6% | | 1,7% |
| *TOTAL* | 4095 | 100,0% | | 273,4% |

*Total answers

**Number of respondents

4. Limitations or issues experienced by patients due to OA

| **Limitations** | **N°** | **Percentage (n=3341)** | **Percentage of cases (n=1455)** |
| --- | --- | --- | --- |
| *Physical activities* | 1325 | 39,7% | 91,1% |
| *Work activities* | 715 | 21,4% | 49,1% |
| *Social interactions* | 539 | 16,1% | 37,0% |
| *Emotional, psychological, or mental health issues* | 400 | 12,0% | 27,5% |
| *Sex life* | 362 | 10,8% | 24,9% |
| *TOTAL* | 3341 | 100,0% | 229,6% |

*Total answers

**Number of respondents
