# Supplementary material for: A patients’ view of OA: the Global Osteoarthritis Patient Perception Survey (GOAPPS), a pilot study
Source: BMC Musculoskelet Disord. 2020 Nov 7;21:727. doi: 10.1186/s12891-020-03741-0 (PMC7648975; doi:10.1186/s12891-020-03741-0)
Supplement: Supplementary file 3 — Additional file 3. Figure showing the joints reported by participants in the category “other”. Patients could report OA in joints other than the options offered as an answer (knee, hip, spine, hand). Each column represents the localization(s) of OA as diagnosed by their medical doctor. [file 12891_2020_3741_MOESM3_ESM.docx]

**Additional file 3**. Joints reported by participants in the category “other”. Patients had the possibility to report OA in joints other than the options offered as an answer (knee, hip, spine, hand). Each column represents the localization(s) of OA as diagnosed by their medical doctor.
