# Supplementary material for: A patients’ view of OA: the Global Osteoarthritis Patient Perception Survey (GOAPPS), a pilot study
Source: BMC Musculoskelet Disord. 2020 Nov 7;21:727. doi: 10.1186/s12891-020-03741-0 (PMC7648975; doi:10.1186/s12891-020-03741-0)
Supplement: Supplementary file 4 — Additional file 4. Table showing participants’ responses to questions related to the causes of OA and their relationship with their doctor. [file 12891_2020_3741_MOESM4_ESM.docx]

**Additional file 4**. Participants' responses to questions related to the causes of OA and their relationship with their doctor.

| **Questions** | **Strongly agree** | **Agree** | **Neither agree nor disagree** | **Disagree** | **Strongly disagree** |
| --- | --- | --- | --- | --- | --- |
| ***7. I understand the common causes of osteoarthritis.*** *(n=1485)* | 18.3% (n=272) | 50.2% (n=746) | 18.5% (n=275) | 9.2% (n=136) | 3.8% (n=56) |
| ***8. My doctor understands me when I describe the symptoms, I experience due to osteoarthritis.*** *(n=1484)* | 19.4% (n=288) | 51.4% (n=763) | 16.3% (n=242) | 8.3% (n=123) | 4.6% (n=68) |
| ***9. My doctor adequately explained my osteoarthritis diagnosis to me.*** *(n=1475)* | 15.7% (n=231) | 42.6% (n=629) | 19.6% (n=289) | 17.4% (n=256) | 4.7% (n=70) |
| ***10. I understand my osteoarthritis treatment options and the associated risks of each option.*** *(n=1468)* | 11.5% (n=169) | 41.8% (n=613) | 22.2% (n=326) | 18.7% (n=274) | 5.9% (n=86) |
| ***11. I am satisfied with my current osteoarthritis treatment plan.*** *(n=1463)* | 5.7% (n=83) | 21.1% (n=308) | 31.0% (n=453) | 28.2% (n=412) | 14.1% (n=207) |
