# Supplementary material for: A patients’ view of OA: the Global Osteoarthritis Patient Perception Survey (GOAPPS), a pilot study
Source: BMC Musculoskelet Disord. 2020 Nov 7;21:727. doi: 10.1186/s12891-020-03741-0 (PMC7648975; doi:10.1186/s12891-020-03741-0)
Supplement: Supplementary file 5 — Additional file 5. Table reporting the limitations experienced due by OA as reported. [file 12891_2020_3741_MOESM5_ESM.docx]

**Additional file 5.** Limitations experienced due by OA as reported

| **Which of the following limitations or issues**  **have you experienced due to osteoarthritis?** | **No** | **Percentage** | | |
| --- | --- | --- | --- | --- |
| Emotional, psychological, or mental health issues | 0,7 | | 10 |  |
| Limitations to physical activities | 29,3 | | 427 |  |
| Limitations to physical activities, Limitations on work activities | 0,8 | | 11 |  |
| Limitations to physical activities, Limitations to social interactions | 0,2 | | 3 |  |
| Limitations to physical activities, Emotional, psychological, or mental health issues | 4,9 | | 71 |  |
| Limitations to physical activities, Limitations to sex life | 2,3 | | 33 |  |
| Limitations to physical activities, Limitations to sex life, Emotional, psychological, or mental health issues | 1,4 | | 20 |  |
| Limitations to physical activities, Limitations to social interactions | 4,9 | | 71 |  |
| Limitations to physical activities, Limitations to social interactions, Emotional, psychological, or mental health issues | 3,7 | | 54 |  |
| Limitations to physical activities, Limitations to social interactions, Limitations to sex life | 1,5 | | 22 |  |
| Limitations to physical activities, Limitations to social interactions, Limitations to sex life, Emotional, psychological, or mental health issues | 1,1 | | 16 |  |
| Limitations to physical activities, Limitations to social interactions, Limitations to work activities | 6,6 | | 96 |  |
| Limitations to physical activities, Limitations to social interactions, Limitations to work activities, Emotional, psychological, or mental health issues | 4,4 | | 64 |  |
| Limitations to physical activities, Limitations to social interactions, Limitations to work activities, Limitations to sex life | 2,9 | | 42 |  |
| Limitations to physical activities, Limitations to social interactions, Limitations to work activities, Limitations to sex life, Emotional, psychological, or mental health issues | 11,4 | | 166 |  |
| Limitations to physical activities, Limitations to work activities | 10,5 | | 153 |  |
| Limitations to physical activities, Limitations to work activities, Emotional, psychological, or mental health issues | 5,8 | | 84 |  |
| Limitations to physical activities, Limitations to work activities, Limitations to sex life | 1,8 | | 26 |  |
| Limitations to physical activities, Limitations to work activities, Limitations to sex life, Emotional, psychological, or mental health issues | 1,0 | | 15 |  |
| Limitations to physical activities, Limitations to work activities, Limitations to sex life, Emotional, psychological, or mental health issues | 1,0 | | 15 |  |
| Limitations to sex life | 0,1 | | 2 |  |
| Limitations to sex life, Emotional, psychological, or mental health issues | 0,1 | | 1 |  |
| Limitations to social interactions | 0,1 | | 2 |  |
| Limitations to social interactions, Emotional, psychological, or mental health issues | 0,5 | | 8 |  |
| Limitations to social interactions, Limitations to work activities | 1,4 | | 20 |  |
| Limitations to social interactions, Limitations to work activities, Emotional, psychological, or mental health issues | 0,1 | | 1 |  |
| Limitations to social interactions, Limitations to work activities, Limitations to sex life | 0,1 | | 1 |  |
| Limitations to work activities | 1,2 | | 18 |  |
| Limitations to work activities, Limitations to sex life | 0,1 | | 2 |  |
| Limitations to work activities, Limitations to sex life, Emotional, psychological, or mental health issues | 0,1 | | 1 |  |
| TOTAL | 1455 | | 100 |  |
